# Recycling Lithium from Waste Lithium Bromide to Produce Lithium Hydroxide

**DOI:** 10.3390/membranes11100759

**Published:** 2021-09-30

**Authors:** Wenjie Gao, Xinlai Wei, Jun Chen, Jie Jin, Ke Wu, Wenwen Meng, Keke Wang

**Affiliations:** 1 Collaborative Innovation Center for Environmental Pollution Control and Ecological Restoration of Anhui Province, School of Biology, Food and Environment, Hefei University, Hefei 230601, China; wenjieg96@163.com (W.G.); chenjun@hfuu.edu.cn (J.C.); amushui@hfuu.edu.cn (J.J.); wuke@hfuu.edu.cn (K.W.); mww18205694264@163.com (W.M.); kk954955@163.com (K.W.); 2 Anhui Key Laboratory of Sewage Purification and Eco-Restoration Materials, Hefei 230088, China

**Keywords:** lithium bromide, BMED, recovery, LiOH, clean production

## Abstract

Lithium resources face risks of shortages owing to the rapid development of the lithium industry. This makes the efficient production and recycling of lithium an issue that should be addressed immediately. Lithium bromide is widely used as a water-absorbent material, a humidity regulator, and an absorption refrigerant in the industry. However, there are few studies on the recovery of lithium from lithium bromide after disposal. In this paper, a bipolar membrane electrodialysis (BMED) process is proposed to convert waste lithium bromide into lithium hydroxide, with the generation of valuable hydrobromic acid as a by-product. The effects of the current density, the feed salt concentration, and the initial salt chamber volume on the performance of the BMED process were studied. When the reaction conditions were optimized, it was concluded that an initial salt chamber volume of 200 mL and a salt concentration of 0.3 mol/L provided the maximum benefit. A high current density leads to high energy consumption but with high current efficiency; therefore, the optimum current density was identified as 30 mA/cm^2^. Under the optimized conditions, the total economic cost of the BMED process was calculated as 2.243 USD·kg^−1^LiOH. As well as solving the problem of recycling waste lithium bromide, the process also represents a novel production methodology for lithium hydroxide. Given the prices of lithium hydroxide and hydrobromic acid, the process is both environmentally friendly and economical.

## 1. Introduction

Lithium is the lightest alkali metal and shows excellent performance and wide applicability in batteries, ceramics, glass, and lithium-based lubricating oils, among other things [1,2]. In recent years, the world has witnessed a tremendous increase in the consumption of lithium, stimulated especially by the development of new electric vehicles. It is estimated that the lithium battery market will reach USD 50 billion by 2025 [3], with global lithium consumption reaching about 498,000 tons [4]. The huge market share of lithium-ion batteries (LIBs) further intensifies the lithium supply risks. This makes efficient recycling of lithium resources an inevitable requirement [5].

As a downstream product of the lithium industry, lithium bromide is generally synthesized from hydrobromic acid and lithium carbonate. Lithium bromide is easily soluble in water and exhibits a very strong water imbibition tendency; therefore, it is commonly used as the working medium in various drying systems [6]. Lithium bromide is most widely used in industry as a water-vapor-absorbent material and an air humidity regulator [7,8]. At present, most absorption refrigerators operate by using lithium bromide as the absorbent and water as the refrigerant [9]. However, there are few studies on the recovery of lithium bromide after its use in the above processes. In view of the likelihood of lithium resource shortages in the future, novel methods for the efficient recovery of lithium bromide need to be developed. Lithium hydroxide is the most important lithium chemical, finding wide application in a variety of lithium industries and downstream lithium products [10]. After the impact of COVID-19 in 2020, the price of lithium hydroxide began to rise rapidly in 2021. At present, the main method adopted for the industrial production of lithium hydroxide involves roasting spodumene with sulfuric acid, then adding sodium hydroxide and soda ash to neutralize the excess sulfuric acid and remove impurities. Finally, this is converted into lithium hydroxide [11,12]. The limitations of this method are that it consumes large amounts of raw materials and generates low-purity sodium sulfate, which needs to be processed further. These factors lead to an increase in the cost of the operation. Therefore, the development of an efficient and eco-friendly method for the conversion of waste lithium bromide into lithium hydroxide could not only solve the problem of the environmental hazards of the waste lithium bromide but also establish a novel economic production process for lithium hydroxide by converting the downstream waste lithium bromide back into the upstream lithium hydroxide.

Electrodialysis (ED) is a membrane separation process based on an ion-exchange membrane [13]. Under the action of an external electric field, the charged ions in the solution move toward the oppositely charged electrode through the ion-exchange membrane to achieve separation, dilution, and concentration of the solution [14,15]. Due to its excellent performance, ED is widely used in wastewater treatment, resource recovery, and desalination of seawater in certain specific processes, etc. [16,17,18]. Bipolar membrane electrodialysis (BMED) is an advanced version of the traditional electrodialysis technology [19]. It combines the benefits of electrodialysis technology and bipolar membranes. The bipolar membrane is a composite anion-exchange membrane and cation-exchange membrane. It is not permeable to any ions; instead, water can be split in the middle layer of the bipolar membrane to produce hydrogen ions and hydroxide ions. However, unlike when water splits at an electrode, no gas is produced [20]. The incorporation of the bipolar membrane into the electrodialysis device can combine the original anions and cations in the solution with the protons and hydroxyl groups produced via the splitting of water, respectively, to generate the corresponding acids and bases [21,22]. Compared with traditional acid and alkali production, the BMED process has the advantages of having no by-product formation, a high energy utilization, and a convenient operational procedure [23,24]. Therefore, BMED has been widely used in chemical synthesis, environmental protection, and food industries [25,26,27].

Because of these advantages, we tried to establish a bipolar membrane electrodialysis system to recover lithium bromide waste. In this system, the lithium ions pass through the cation-exchange membrane and the bromide ions pass through the anion-exchange membrane to combine with the hydroxide ions and protons produced in the bipolar membrane, respectively, forming lithium hydroxide and hydrobromic acid and thus achieving the conversion of lithium bromide to lithium hydroxide. In this paper, we aim to explore the feasibility of the treatment of lithium bromide using BMED and to estimate the factors that affect the performance of the BMED system to achieve an optimized treatment effect. Though the main focus of the study is on the effective treatment of lithium bromide waste, a novel production process for lithium hydroxide could also be developed. An added advantage is that the expensive reagent, hydrobromic acid, is obtained as a by-product during the process. Finally, an economic estimation is performed to give more insight into the advantages of the bipolar membrane electrodialysis technology in enhancing the process outcome.

## 2. Experiments

### 2.1. Materials

The salt chamber solution was lithium bromide of varying concentrations, and the electrode chamber was filled with a 0.3 mol/L sodium sulfate solution. To ensure the smooth functioning of the BMED system, a certain amount of hydrobromic acid and lithium hydroxide was added to the acid chamber and alkali chamber, respectively, at the beginning of the experiments. All chemical reagents used were of analytical grade and were purchased from China National Pharmaceutical Group Co., Ltd., Shanghai, China. The anion-exchange membrane (AMX), cation-exchange membrane (CMX), and bipolar membrane (BP-1) used in the experiments were all purchased from ASTOM Corp., Tokyo, Japan. The main characteristics of the membranes used are shown in Table 1.

### 2.2. Experimental Set-Up

**Figure 1:**
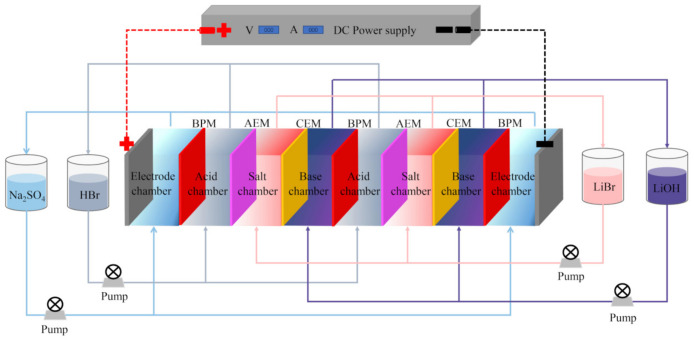
Schematic representation of experimental equipment.

**Figure 2:**
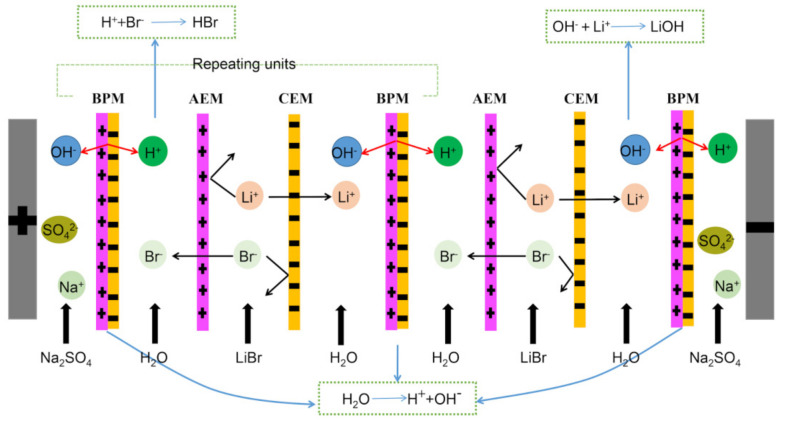
Schematic representation of the BMED stack used in the experiment.

Home-made membrane stacks were used for the experiments. Figure 1 shows a schematic representation of the entire membrane stack device, and another schematic diagram of the BMED stack is displayed in Figure 2. The membrane stack consisted of two anion-exchange membranes, two cation-exchange membranes, three bipolar membranes, and two electrode plates, stacked together as two sets of repeating units. The compartments between two adjacent membranes were separated by 10 mm spacers, and the effective area of each piece of membrane was 18 cm^2^. The electrode plates were titanium plates coated with ruthenium. The entire membrane stack comprised two electrode chambers, two acid chambers, two alkali chambers, and two salt chambers. The chamber connections were based on preprocessing experiments and on previous work [28]. In all the experiments, the initial volume of each of the acid and alkali chambers was 200 mL, and that of each of the electrode chambers was 300 mL. Except in the “effect of initial salt chamber volume” part of the investigation, the initial volume of each of the salt chambers was also 200 mL. In the “effect of initial salt chamber volume” part of the investigation, the salt chamber volume was varied (100–300 mL) to explore the impact on the performance of the BMED process. Four peristaltic pumps (Baoding Lead Fluid Technology Co. Ltd., Baoding, China) were used to pump the feed liquid into the corresponding compartments at a linear velocity of 0.33 m/s to form four circulating loops. A DC power supply (WYL1703, Hangzhou Siling Electrical Instrument Co. Ltd., Hangzhou, China) was connected to the electrode plate to provide the current. The maximum voltage applied to the membrane stack was 40v. The voltage and current values were read directly from the mains. The sSalt chamber conductivity was measured using a portable conductivity meter (DDBJ-350, INESA Scientific Instrument Co. Ltd., Shanghai, China). The temperature in all experiments was kept below 30 °C. Before applying a current to the membrane stack, the initial cycle consisted of eliminating all visible bubbles, and the experiment was stopped when the recovery rate reached 99%.

### 2.3. Analysis and Calculation

The concentrations of lithium hydroxide and hydrobromic acid were determined by titration using a continuous digital titrator (Continuous RS, VITLAB, Muhltal, Germany) with phenolphthalein as the indicator.

The energy consumption, *E* (kWh/kg of LiOH), was calculated using Formula (1) [29]:

(1) $$E=\int_{0}^{t}\frac{UIdt}{C_{t}V_{t}M}$$

where U (V) is the membrane stack voltage, I (A) is the applied current, $C_{t}$ (mol/L) and $V_{t}$ (mL) are the concentration and volume of the lithium hydroxide, respectively, at time t, and M is the molecular weight of lithium hydroxide (M = 23.94834 g·mol^−1^).

The current efficiency (η, %) of the BMED process was calculated using Formula (2) [29]:

(2) $$\eta =\frac{Z\left(C_{t}V_{t}-C_{0}V_{0}\right)F}{NIt}\times 100\%$$

where $C_{t}$ (mol/L) and $V_{t}$ (mL) are the concentration and volume of the lithium hydroxide, respectively, at time t,$\;C_{0}$ (mol/L) and $V_{0}$ (mL) are the concentration and volume of the lithium hydroxide, respectively, at time 0, Z is the ion’s absolute valence (Z = 1 for lithium hydroxide), F is the Faraday constant (96,485 C·mol^−1^), N is the repeating unit number (N = 2) in the BMED stack, I (A) is the applied current, and t (h) is the test time.

## 3. Results and Discussion

### 3.1. Effect of Current Density

The current density is the most important factor affecting the energy consumption and the efficiency of BMED processes, so a current density in the range of 10–50 mA/cm^2^, commonly used in BMED processes, was selected for use in the research [28,30], and the initial concentration of the salt chamber was fixed at 0.3 mol/L according to the pretreatment.

**Figure 3:**
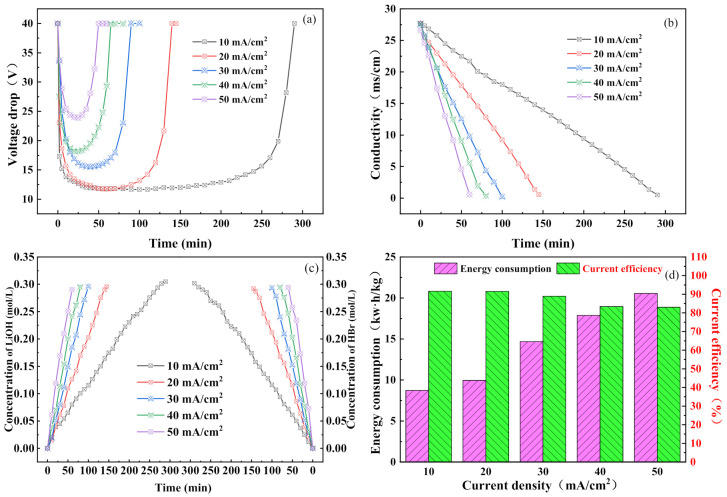
Effect of current density on BMED performance: (**a**) BMED stack voltage drop; (**b**) the conductivity of the salt chamber; (**c**) hydrobromic acid concentration in the acid chamber and lithium hydroxide concentration in the alkali chamber; (**d**) energy consumption and current efficiency of the whole process.

Figure 3a shows the variation in the voltage drop of the membrane stack with time for different current densities. It can be seen that when the current was first applied to the membrane stack, the voltage dropped sharply due to the splitting of water in the bipolar membrane. As the reaction progressed, the voltage of the membrane stack gradually reached a stable state, and at this time the chambers in the membrane stack attained a state of equilibrium. At the end of the experiment, the components in the salt chamber were exhausted and the voltage gradually increased and returned to 40v. Figure 3b illustrates the variation in the electrical conductivity of the salt chamber with time for different current densities. It can be seen that the conductivity of the salt chamber could be reduced to below 500 uS/cm for all the current density values shown. The conversion rate of the salt chamber solution reached 99%, indicating that the lithium bromide in the salt chamber could be converted into hydrobromic acid and lithium hydroxide in all cases [28]. In Figure 3c, it can be seen that as the reaction progressed, the concentrations of the hydrobromic acid in the acid chamber and the lithium hydroxide in the alkali chamber gradually increased. As shown in Figure 2, when a current was applied to the membrane stack, the water in the bipolar membrane was split to produce hydrogen ions and hydroxide ions [20]. The lithium ions in the salt chamber passed through the cation-exchange membrane and combined with the hydroxide ions in the alkali chamber to form lithium hydroxide [31,32]. At the same time, the bromide ions passed through the anion-exchange membrane and combined with the hydrogen ions in the acid chamber to form hydrobromic acid. In addition, with an increase in current density, the concentrations of the acid and base gradually increased. This is because when the current is gradually increased, the splitting of water in the bipolar membrane is accelerated [33]. Thus, a high current density can lead to both higher acid and higher alkali concentrations, which is in agreement with the second Wien effect [34]. It should be pointed out that in the final result, the concentration in the base chamber was slightly higher than that in the acid chamber despite the fact that the same number of protons and hydroxyl groups were produced in the bipolar membrane. This anomaly can be explained by the difference in the ion-exchange membranes. Hydrogen ions pass through the anion-exchange membrane relatively easily compared with the resistance to the flow of hydroxide ions offered by the cation-exchange membrane [35]. This phenomenon is also one of the reasons for the loss in the current efficiency, as depicted in Figure 3d. In this figure, it can be seen that the current efficiency decreased from 91.61% at 10 mA/cm^2^ to 83.02% at 50 mA/cm^2^. A high current led to the intensification of ion reverse diffusion in the later stage of the experiment, resulting in a loss in the current efficiency, which is in line with most research results [36,37,38,39]. In contrast, the energy consumption gradually increased with an increase in the current density. The main reason for this is that the increase in the current density led to the need to overcome a larger membrane stack resistance and consequently the consumption of more ohmic energy. In short, the effect of the current density on the performance of the BMED process was clear. The best “trade-off” between capital cost and operating cost could be achieved when the current density was 30–40 mA/cm^2^. In actual industrial production, it is necessary to balance the current density issues against actual needs to procure the maximum benefits.

### 3.2. Effect of Feed Concentration

Apart from the current density, the initial feed concentration is one of the most important factors influencing the performance of the BMED process. We chose concentrations in the range of 0.1–0.5 for use in this research. A current density of 30 mA was selected, according to the results in Section 3.1.

**Figure 4:**
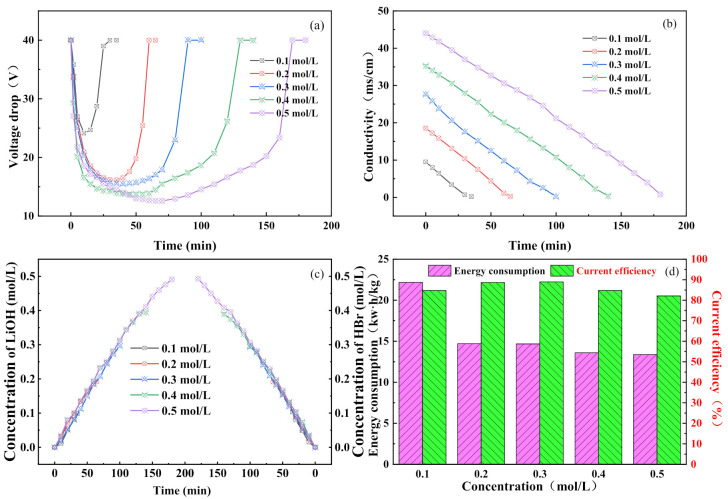
Effect of feed concentration on BMED performance: (**a**) BMED stack voltage drop; (**b**) the conductivity of the salt chamber; (**c**) hydrobromic acid concentration in the acid chamber and lithium hydroxide concentration in the alkali chamber; (**d**) energy consumption and current efficiency of the whole process.

As shown in Figure 4a, the voltage drop in the membrane stack varied with time for different feed concentrations. It can be seen that the voltage drop in the membrane stack first decreased, then reached a stable state, and finally rose sharply. This observation is in line with the results shown in Figure 3a. Clearly, for the various experiments under the same conditions, a longer reaction time was required as the concentration increased. Similarly, as high feed concentrations result in more ions, the voltage drop in the membrane stack was lower at high concentrations. This corresponds to the change in the conductivity of the salt chamber with time for different feed concentrations (Figure 4b). A high concentration implies high conductivity and requires a longer reaction time. Under the same current density conditions, the conversion rate of lithium bromide to lithium hydroxide and hydrobromic acid in the salt chamber was also the same. The conductivity of the salt chamber at all concentrations eventually dropped below 500 uS/cm. Figure 4c shows the time-varying curves for the hydrobromic acid in the acid chamber and the lithium hydroxide in the base chamber under different feed concentrations. As the reaction progressed, the concentrations of the acid and base gradually increased, which indicates the success of the reaction and proves the feasibility of the experimental scheme. A high feed concentration eventually resulted in high concentrations of the acid and base. The final results showed that the lithium bromide conversion rate reached values of up to 99% for all feed concentrations. Figure 4d shows the current efficiency and the energy consumption at different feed concentrations. It can be seen that the current efficiency decreased with an increase in feed concentration. This phenomenon can be explained by considering the following three aspects. Firstly, according to Donnan’s equilibrium theory [40,41], a high electrolyte concentration reduces the selectivity of a monopolar ion-exchange membrane, increases the migration of co-ions, and consequently reduces the current efficiency. Secondly, a high feed concentration leads to the diffusion of molecules in the salt chamber to the acid and base chambers, resulting in a loss of current efficiency. Finally, with a high salt concentration, the osmotic pressure around the bipolar membrane increases, making it difficult for water molecules to migrate to the bipolar membrane. This causes the rate of splitting of water to decrease and the current efficiency to gradually decrease, contributing to a decrease in the efficiency of the synthesis. Concentration polarization may be an additional cause of this phenomenon [42]. The energy consumption decreased gradually with the increase in feed concentration, especially between 0.1 mol and 0.2 mol; this is because an increase in electrolyte concentration greatly reduces the membrane stack resistance. A low energy consumption and a high current efficiency maximize the benefits; hence, we identified 0.3 mol/L as the ideal concentration.

### 3.3. Effect of Initial Salt Chamber Volume

To further optimize the performance of the treatment methodology under study, we attempted to examine the effect of the initial salt chamber volume. The salt chamber volume was chosen within the range of 100–300 mL. The optimum current density and the optimum initial salt chamber concentration were already identified as 30 mA/cm^2^ and 0.3 mol/L, respectively.

**Figure 5:**
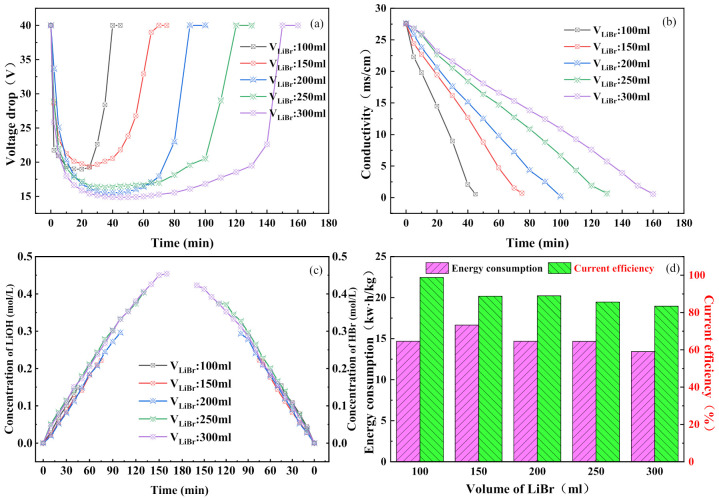
Effect of initial salt chamber volume on BMED performance: (**a**) BMED stack voltage drop; (**b**) the conductivity of the salt chamber; (**c**) hydrobromic acid concentration in the acid chamber and lithium hydroxide concentration in the alkali chamber; (**d**) energy consumption and current efficiency of the whole process.

As shown in Figure 5a, the voltage drop in the membrane stack varied with time for the different salt chamber volumes. The trend is the same as that observed in Figure 3a and Figure 4a. When an electric current is applied to the bipolar membrane, water molecules are split into hydrogen ions and hydroxide ions, which significantly reduces the resistance of the membrane stack. Figure 5b shows the changes in the conductivity of the salt chamber for different salt chamber volumes. It can be seen that as the volume of the salt chamber increased, the rate of decrease in the conductivity became less and the time required for the reaction became longer [43]. All salt chamber volumes resulted in complete conversion. Figure 5c shows the concentration of hydrobromic acid in the acid chamber and of lithium hydroxide in the alkali chamber for different salt chamber volumes. It can be seen that as the reaction progressed, all experimental concentrations gradually increased. This shows that the hydrogen ions and the hydroxide ions passed through the cation- and anion-exchange layers of the bipolar membrane, respectively, to reach the acid chamber and the alkali chamber, and successfully combined with the bromide and lithium ions to form hydrobromic acid and lithium hydroxide [44]. The results suggest that the larger the volume of the salt chamber, the higher the concentrations of the acid and base generated. Figure 5d shows the energy consumption and the current efficiency for different salt chamber volumes. It can be seen that the current efficiency gradually decreased with an increase in the salt chamber volume, which was mainly due to back diffusion, unnecessary water splitting, and increased water penetration [45]. This is also the reason for the increase in energy consumption observed at 100–150 mL. As the volume of the salt chamber continued to increase, the conversion of lithium bromide increased and the specific energy consumption decreased, resulting in an overall decrease in energy consumption [46]. Considering the energy consumption and the current efficiency comprehensively, it was identified that when the volume of the salt chamber was 200 mL, that is, when the volume ratio of the salt chamber to the acid (alkali) chamber was 1:1, the optimum performance of the BMED process could be achieved.

### 3.4. Economic Analysis

The study shows that it is feasible to use BMED to process waste lithium bromide to produce lithium hydroxide, and the relevant experimental conditions have been optimized. To further illustrate the superiority of the methodology and to provide a reference for future industrial applications, we estimated the economic benefits of using BMED to process lithium bromide.

Table 2 shows the economic estimation for the BMED process. It should be pointed out that the following table (Table 2) is based on a current density of 30 mA/cm^2^, a salt chamber volume of 200 mL, and a salt chamber concentration of 0.3 mol/L, as these values were identified as the optimum reaction conditions in the present study. The calculation method was based on the previous literature [47,48], and the annual processing capacity was calculated on the basis of 8720 h. The total process cost is the total energy cost plus the total fixed cost. The total fixed cost is the cost of the membrane stack plus the cost of the peripheral equipment. The cost of the peripheral equipment is 1.5 times the cost of the membrane stack, which, in turn, is 1.5 times the membrane cost. The amortization of the membranes and the peripheral equipment was calculated for 3 years, with an annual interest rate of 8%. The equipment maintenance cost was calculated at 10% of the total investment cost. Electricity charges were calculated as per China’s electricity prices. The total process cost was calculated as 2.243 USD·kg^−1^ LiOH. In contrast, the traditional acid roasting process costs more than 5 USD·kg^−1^ LiOH [49]. This process produces lithium hydroxide by recycling waste lithium bromide as the raw material and obtains hydrobromic acid as a by-product. Considering the expensive nature of both lithium hydroxide and hydrobromic acid, this process is considered to be very economical. In addition, a novel method for processing waste lithium bromide is demonstrated in this study, with the generation of no harmful by-products. Overall, the process strikes a balance between being economical and environmentally friendly, with good development prospects.

## 4. Conclusions

Bipolar membrane electrodialysis technology was used to treat waste lithium bromide. It allowed the conversion of lithium bromide into lithium hydroxide and hydrobromic acid without the need for other reagents. The effects of the current density, the feed salt concentration, and the initial salt chamber volume on the BMED process performance were estimated by investigating the concentrations of lithium hydroxide and hydrobromic acid, the current efficiency, the energy consumption, and other indicators. The current density was the most important factor affecting the performance of the BMED process. A high current density increases the processing capacity of the BMED process but has the limitation of consuming more energy. The lowest energy consumption of 8.73 kWh·kg^−1^ LiOH was obtained when the current density was 10 mA/cm^2^, and an optimal current density of 30 mA/cm^2^ was selected through a comprehensive evaluation. The higher the feed salt concentration and the initial salt chamber volume, the lower the energy consumption, but at the same time a certain amount of current efficiency will be lost in overcoming the back diffusion of the membrane stack. The total cost of the BMED process for converting lithium bromide into lithium hydroxide was estimated to be 2.243 USD·kg^−1^ LiOH, and the conversion rate of lithium bromide reached a maximum of 99%. The study investigated the scope of the BMED process and identified it as a promising novel approach for the recycling of waste lithium bromide into valuable synthetic reagents, thereby reducing its detrimental effects on the environment.

## Figures and Tables

**Table 1:** Properties of the membranes used in the BMED experiments ^a^.

Membrane Characteristics	AMX	CMX	BP-1
IEC (meq·g^−1^)	1.4–1.7	1.5–1.8	-
Thickness (µm)	120–180	220–260	200–350
Area resistance (Ω·cm^2^)	2.0–3.5	2.0–3.5	-
Voltage drop (V)	-	-	1.2–2.2
Current efficiency (%)	-	-	>98
Transport number (%)	91	98	>98

^a^ Data from the manufacturer’s instruction manual.

**Table 2:** Economic analysis of BMED treatment of lithium phosphate.

Parameters	BMED Process
Feed volume (L)	0.2
Feed salt concentration (g·L^−1^)	26.055
Current density (mA·m^−2^)	30
Batch experiment time (h)	1.67
Effective each membrane area (cm^2^)	18
Energy consumption (kWh·kg^−1^ LiOH)	14.672
Treatment capacity (kg LiOH·year^−1^)	13.6
Price of bipolar membrane (USD·m^−2^)	800
Price of mono membrane (USD·m^−2^)	200
Membrane lifetime and amortization of the peripheral equipment (year)	3
Electricity charge (USD·kWh^−1^)	0.0825
Membrane cost (USD)	5.76
Membrane stack cost (USD)	8.64
Peripheral equipment cost (USD)	12.96
Total investment cost (USD)	27.36
Amortization (USD·year^−1^)	9.12
Interest (USD·year^−1^)	2.1888
Maintenance (USD·year^−1^)	2.736
Total fixed cost (USD·year^−1^)	14.0448
Total fixed cost (USD∙kg^−1^ LiOH)	1.033
Energy cost (USD·kg^−1^ LiOH)	1.21
Total process cost (USD·kg^−1^ LiOH)	2.243

## Data Availability

Not applicable.
